# What’s New in the Diagnosis of Periprosthetic Joint Infections: Focus on Synovial Fluid Biomarkers

**DOI:** 10.3390/tropicalmed7110355

**Published:** 2022-11-07

**Authors:** Giuseppe Solarino, Davide Bizzoca, Lorenzo Moretti, Giovanni Vicenti, Andrea Piazzolla, Biagio Moretti

**Affiliations:** 1 Department of Basic Medical Sciences, Neuroscience and Sense Organs, Orthopaedic and Trauma Unit, University of Bari “Aldo Moro”-AOU Consorziale Policlinico di Bari, Piazza Giulio Cesare, 11, 70124 Bari, Italy; 2 AOU Consorziale Policlinico di Bari, UOSD Spinal Surgery and Scoliosis Deformity Centre, Piazza Giulio Cesare, 11, 70124 Bari, Italy

**Keywords:** PJI, synovial fluid, proteome, metabolome, omics sciences, THA, TKA, THR, TKR

## Abstract

Periprosthetic joint infections are some of the leading causes of revision prosthetic surgery, accounting for 25% of failed total knee replacements and 15% of failed total hip replacements. The search for a biomarker that, together with clinical and radiological findings, could improve the management of such patients is currently a significant challenge for orthopaedic surgeons. Synovial fluid is a viscous and mucinous substance produced by the synovium, a specialized connective tissue that lines diarthrodial joints. Synovial fluid is an ultrafiltrate of plasma but also contains proteins secreted from the surrounding tissues, including the articular cartilage and synovium. Therefore, synovial fluid represents a source of disease-related proteins that could be used as potential biomarkers in several articular diseases. Based on these findings, the study of synovial fluid has been gaining increasing importance in recent years. This review aims to assess the accuracy and the limitations of the most promising synovial fluid biomarkers—i.e., Alpha-Defensin, Leukocyte Esterase, C-Reactive Protein, Interleukin-6, Calprotectin, Presepsin and Neopterin—in the diagnosis of PJI. Special attention will be given to emerging synovial biomarkers, which could soon be important in diagnosing PJIs.

## 1. Introduction

Periprosthetic joint infections (PJIs) are some of the leading causes of revision prosthetic surgery, accounting for 25% of failed total knee replacements (TKR) and 15% of failed total hip replacements (THR) [1,2,3].

The search for a biomarker that, together with clinical and radiological findings, could improve the management of such patients is currently a significant challenge for orthopaedic surgeons.

In this context, the study of synovial fluid biomarkers might play a central role in future research settings and daily clinical practice.

Compared with plasma, synovial fluid (SF) has the advantage of being near the joint tissues, which are primarily altered during these articular diseases. It can be collected with arthrocentesis, i.e., a minimally invasive articular procedure [4]. Thus, in recent years, the study of SF has been gaining increasing importance in orthopaedics.

This review aims to assess the accuracy and the limitations of the most promising synovial fluid biomarkers—i.e., Alpha-defensin (Alpha-D), Leukocyte Esterase (LE), Interleukin-6 (IL-6), Calprotectin, Presepsin and Neopterin—in the diagnosis of PJI.

Special attention will be given to emerging synovial biomarkers that could play an important role in the diagnosis of PJIs shortly

## 2. Synovial Fluid Physiology and Composition

Synovial fluid (SF) is a viscous and mucinous substance produced by the synovium, a specialized connective tissue that lines diarthrodial joints. Although the main function of SF is to lubricate joints, thus reducing friction between the articular surfaces, it also allows nutrients and catabolites to circulate between the avascular articular cartilage and the vascularized synovial membrane [1].

SF is an ultrafiltrate of plasma, containing the same plasmatic levels of glucose and uric acid. The SF protein concentration, however, is about one-third of the plasmatic one [5]. Plasma constituents that enter joint fluid must cross a double-barrier membrane. First, the endothelial lining of the capillaries is scratched, followed by movement through a matrix that surrounds synovial cells [6]. This ultrafiltrate is finally combined with the hyaluronate synthesized by the synovium [6]. It accumulates in a pathologic joint and reflects the ongoing process of the joint disorder [7].

Therefore, SF might represent a potential source of disease-related proteins that could be used as biomarkers in several articular diseases. Consequently, in recent years, the study of SF has gained an increasing interest in orthopaedics and rheumatology to better investigate the pathogenesis and improve the management of several joint diseases, including osteoarthritis (OA), rheumatoid arthritis (RA) and other autoimmune non-rheumatoid-arthritides, osteochondrosis, articular infection and periprosthetic joint infection (PJI) [4,8,9,10,11,12].

## 3. PJI Definition According to the 2018 International Consensus Meeting (ICM) of Philadelphia

Hip and knee PJI are currently diagnosed according to the 2018 Philadelphia ICM criteria (Table 1) [13]. Two positive cultures or a sinus tract presence are considered major criteria; at least one major criterium is sufficient to diagnose a PJI [13].

In absence of major criteria, minor criteria are considered to diagnose a PJI; in this case, a minimum score of 6 (out of 12) is needed to diagnose a PJI [13]. Minor criteria scoring is defined as follows: 2 points for a serum CRP > 1 mg/dL; 2 points for D-dimer > 860 ng/mL; 2 points for erythrocyte sedimentation rate (ESR) > 30 mm/h; 3 points for a synovial fluid white blood cell count >3000 cells/μL; 3 points for an increased synovial fluid alpha-defensin (signal-to-cut off ratio > 1); 3 points for an elevated synovial fluid leukocyte esterase (++); 3 points for polymorphonuclear percentage > 80%; and 2 points for synovial CRP > 6.9 mg/L. While an aggregate score ≥ 6 defines a PJI, a score between 2 and 5 requires the evaluation of intraoperative findings to confirm or refuse the diagnosis [13].

Intraoperative findings include favourable histology (3 points), purulence (3 points) and single positive culture (2 points). The sum of preoperative and intraoperative scores was finally assessed: a combined score ≥ 6 defines a PJI, a score between 4 and 5 is considered inconclusive and a score ≤ 3 depicts the lack of infection.

## 4. Synovial Fluid Biomarkers in PJIs

Synovial fluid biomarkers and blood cell composition acquired a relevant role, among minor criteria, in the diagnosis of hip and knee PJI according to the ICM 2018 of Philadelphia.

The present paragraph will discuss the main features of the synovial fluid biomarkers recognized by ICM Philadelphia 2018 and emerging synovial fluid biomarkers. As recently stated by Ahmad et al. [14], no synovial biomarker should be applied as a standalone diagnostic tool based on current evidence. Currently, these biomarkers could be used as an adjunct in the diagnostic work-up of PJIs but clinical research should aim at validating new biomarkers to improve PJIs diagnosis in a near future.

### 4.1. Synovial Biomarkers Validated by 2018 ICM Philadelphia

#### 4.1.1. Alpha-Defensin (AD)

Alpha-defensin (AD) is an antimicrobial protein released by neutrophils in response to pathogens [15]. AD then enters the pathogen’s cell membrane and causes its rapid killing, thus supporting the immune system [15].

According to ICM Philadelphia 2018, an elevated synovial fluid AD, defined as signal-to-cut-off ratio >1, is a minor criterium to define PJI diagnosis; hence, without major criteria, AD should be routinely assessed to confirm or rule out a PJI [13].

However, it is important to remark that AD can be detected both by an Enzyme-Linked Immunosorbent Assay (ELISA) laboratory test or by an AD test kit [16]. Ahmad et al. [14], in a recent meta-analysis analysing 42 papers, reported that the laboratory-based AD ELISA test has a high accuracy for PJI diagnosis, whereas the AD test kit showed a markedly lower accuracy. Hence, the laboratory-based AD ELISA test should be preferred in daily clinical practice.

Kuo et al. [17], in a recent retrospective study based on 76 patients who underwent TKA or THA revision surgery, reported that AD, of the minor criteria of 2018 ICM Philadelphia, has the best diagnostic performance, i.e., AUC = 0.92.

#### 4.1.2. Leukocyte Esterase (LE)

Leukocyte Esterase (LE) is an esterase expressed in white blood cells; thus, its presence in a sample indicates leucocyte expression. An elevated synovial fluid LE is a minor criterium used to define PJIs, according to 2018 ICM Philadelphia; it is a diagnostic tool with a 2+ cut-off, and a rapid and inexpensive test [18].

Ahmad et al. [14], in a recent meta-analysis based on 42 clinical studies, demonstrated that synovial LE does not reach a diagnostic accuracy higher than positive culture bacteriology or synovial white cell counts, thus limiting the clinical relevance of this biomarker.

Interestingly, however, Logoluso et al. [19], in a recent retrospective study collecting the data of 76 patients who received a 2-stage exchange for PJI, assessed the reliability of intraoperative synovial fluid LE as compared to the serum CRP and ESR concentrations. These authors reported the LE synovial strip test proved a reliable tool to diagnose the persistence of infection (AUC 0.9044) since it outperformed the serum CRP and ESR assays. Hence, the synovial LE strip test provides a valuable intraoperative diagnostic during second-stage revision for PJI.

Furthermore, Chisari et al. [18], in a retrospective study based on 259 patients undergoing TKA or THA revision surgery, suggested using the cut-off of LE1+ (result = negative or trace) as a point of care test to rule out a PJI, whereas LE at 2 + threshold has near absolute specificity for PJI diagnosis.

#### 4.1.3. C-Reactive Protein (CRP)

C-Reactive Protein (CRP), i.e., a pentameric protein member of the pentraxin family of proteins, is an acute-phase protein synthesized by the liver.

Although the diagnostic utility of synovial fluid CRP concentration has been debated in recent years, according to ICM Philadelphia 2018, a synovial CRP concentration > 6.9 mg/L is a minor criterion for PJI diagnosis.

Baker et al. [20], in a recent study recruiting 621 participants who underwent evaluation for a suspicious PJI before receiving a revision THA or TKA, demonstrated that synovial CRP has excellent accuracy when used to assess the presence of a PJI. These authors, moreover, showed a good correlation between serum and synovial CRP levels in the recruited patients [20]. Based on these findings, the authors support the use of synovial CRP as an adjunct in the workup of PJI.

### 4.2. Emerging Synovial Fluid Biomarkers

In this session emerging synovial fluid biomarkers, not included in the 2018 ICM Philadelphia criteria will be discussed (Table 2). The following biomarkers will be analysed: Interleukin-6 (IL-6); Calprotectin (CLP); Presepsin (PS) and Neopterin (NPT).

#### 4.2.1. Interleukin-6 (IL-6)

IL-6 is a pro-inflammatory cytokine that induces the expression of a variety of proteins responsible for acute inflammation [21].

Yu et al. [21], in a prospective study recruiting 139 patients affected by hip or knee PJI, showed that synovial IL-6 has a higher diagnostic accuracy for PJI, compared with synovial fluid CRP. These authors recommend assessing synovial IL-6 in patients with an increased serum IL-6 [21].

Nonetheless, Quin et al. [22], in a prospective study, recruited 102 patients with joint pain after arthroplasty, i.e., 39 patients with aseptic prosthesis loosening, 26 patients with acute rheumatoid arthritis (RA, n = 26) and 37 patients suffering from PJI, and demonstrated that current synovial IL-6 levels do not accurately rule out the presence of PJI in patients suffering from RA and complaining of a painful THA or TKA.

#### 4.2.2. Calprotectin (CPT)

Calprotectin is cytoplasmatic calcium- and zinc-binding protein expressed mainly in neutrophils; it is released in the extracellular environment following neutrophil activation and exhibits anti-microbial activity [23].

Faecal CPT has been used for years in the diagnosis of inflammatory bowel disease (IBD) [24], but in more recent years it has gained increasing interest in the study and diagnosis of PJI.

It is a cheap and easy-to-use test compared to other biomarkers.

Wouthuyzen-Bakker et al. [23], in a pilot prospective study comparing 19 patients suffering from PJI to 42 control patients, revealed that the calprotectin test could be considered suggestive of PJI when >50 mg/L. This cut-off level showed excellent diagnostic accuracy for PJI with an area under the curve of 0.94, a sensitivity of 89% and a specificity of 90% [23].

Therefore, these authors conclude that synovial CPT may be a valuable biomarker and useful, especially in excluding a PJI [23].

Salari et al. [25], in a prospective observational study recruiting 76 patients, recently investigated the reliability of synovial CPT in PJI, suggesting it has high sensitivity and specificity in the diagnosis of chronic knee PJI.

The same research group [26], in a more recent prospective observational study recruiting 93 patients suffering from painful TKA, compared the sensibility and sensitivity of both the enzyme-linked immunosorbent assay (ELISA) CPT test and the rapid calprotectin test (CalFAST) to leukocyte esterase (LE). These authors demonstrated that the CPT ELISA test and CalFAST had similar sensitivity (92.3% and 97.4%, respectively) and specificity, whereas the LE rapid test showed 46% of sensitivity and 94% of specificity [26]. The authors concluded that synovial CPT has high accuracy in knee PJI diagnosis and both ELISA and rapid tests are valid. Consequently, the CPT rapid test can be considered an excellent point-of-care test in clinical practice [26].

Moreover, Lazic et al. [27] recently investigated the role of synovial CPT in patients suffering from PJI and concomitant non-infectious inflammation due to recent surgery or dislocation or implant breakage in primary and revision TKA and THA. Interestingly, these authors concluded that, even in the presence of local inflammation due to other, non-infectious causes, calprotectin is a reliable diagnostic parameter for the detection of a PJI.

Finally, Hantouly et al. [28], in a recent meta-analysis involving 618 patients from eight studies, observed that synovial CPT has a pooled sensitivity of 92% and a specificity of 93% in the diagnosis of PJI [28]. Hence, synovial CPT is a valuable biomarker as it provides a reliable and rapid diagnosis of PJI. This emerging biomarker can be used in clinical practice due to its high sensitivity and specificity, comparable to the other utilized biomarkers [27]. In another meta-analysis, similar results were reported by Jisi et al. in [28], thus synovial CPT could be a diagnostic criterion for PJI in a near future.

#### 4.2.3. Presepsin (PS)

Presepsin, i.e., the N-terminal fragment of the soluble cluster of differentiation 14-SubType (sCD14-ST), is released into circulation after the activation of defence mechanisms, mainly bacterial phagocytosis [29,30,31].

This biomarker, originally studied and validated in the diagnosis and prognosis stratification of sepsis [32], has recently been proposed as a potential biomarker for the study of PJI and septic arthritis [29,30,31,32,33].

Imagama et al. [34] recently evaluated synovial fluid and serum PS and procalcitonin (PCT) levels in 18 patients with septic arthritis (SA), compared with 28 patients affected by osteoarthritis (OA), to determine whether presepsin would be useful in the diagnosis of SA.

These authors observed that synovial fluid, blood presepsin and blood PCT were significantly higher in the SA group than in the OA group. Synovial fluid presepsin exhibited both 100% sensitivity and 100% specificity in the SA group, which were at higher rates than those for blood presepsin and PCT. Thus, Imaga et al. concluded that synovial fluid presepsin could be a new biomarker of septic arthritis [34].

Nonetheless, recent studies [34,35] have limited the diagnostic and prognostic role of synovial fluid PS in PJI, compared to serum presepsin concentration, therefore future studies with larger samples are needed to better define the role of synovial PS concentration in PJI diagnosis.

#### 4.2.4. Neopterin (NPT)

Neopterin (NPT), a member of the pteridine family, is a catabolic product of guanosine triphosphate via guanosine triphosphate cyclohydrolase and is released from macrophages following T-cell-dependent interactions involving interferon-γ (IFN-γ). Therefore, it indicates a pro-inflammatory status and serves as a marker of cellular immune system activation [35,36].

Busch et al. [35], in a prospective cohort study recruiting 80 patients with painful hip, shoulder and knee arthroplasty, evaluated the synovial fluid NPT, PS and TNF-α as diagnostic parameters. These authors observed synovial fluid NPT was 59% specific and 74% sensitive with a cut-off value of 7.2 nmol/L, whereas the sensitivity and specificity of synovial fluid TNF-α were 63 and 51%, with a cut-off value of 3.9 pg/mL, and the synovial fluid presepsin was 51% specific and 29% sensitive, with a cut-off value above 0.06 ng/mL [35].

Based on these findings, synovial fluid NPT was found to be a reliable diagnostic marker for the detection of PJI, while synovial fluid TNF-α and PS were too inconsistent to exclude or diagnose PJI.

**Table 2:** Main data of studies focusing on emerging synovial fluid biomarkers for PJI diagnosis.

Study	Design	Sample Size	Main Findings
Yu et al. [21]	Prospective study	139 patients with PJI	Synovial IL-6: higher diagnostic accuracy than CRP
Quin et al. [22]	Prospective study	102 patients with PJI	Synovial IL-6 levels do not accurately rule out the presence of PJI, in patients suffering from RA
Salari et al. [25]	Prospective study	76 patients with PJI	Synovial CPT: high sensibility and specificity in the diagnosis of PJI
Grassi et al. [26]	Prospective study	93 patients with PJI	Synovial CPT ELISA test and CalFAST had similar sensitivity and specificity
Imagama et al. [34]	Prospective study	18 patients with SA, vs. 28 patients with OA	Synovial PS useful in the diagnosis of SA
Busch et al. [35]	prospective study	80 patients	Synovial NPT was 59% specific and 74% sensitive for PJI

CRP = C-Reactive Protein; RA = Rheumatoid Arthritis; CPT = Calprotectin; SA = Septic Arthritis; OA = Osteoarthritis; NPT = Neopterin.

## 5. Discussion

The diagnosis of PJI is currently based on ICM Philadelphia 2018 guidelines. Nonetheless, in daily clinical practice, decision-making in patients with a suspected PJI could be challenging [1,3]. Hence, the search for synovial fluid biomarkers that could improve the diagnosis of PJIs has gained increasing importance in recent years [37,38].

In 2021, the European Bone and Joint Infection Society (EBJIS) published a novel three-level approach to the diagnosis PJIs, which was revealed to be useful in reducing the number of uncertain diagnoses, allowing easier clinical decision-making [39,40]. Although EBJIS guidelines consider several data that were excluded from ICM Philadelphia 2018 guidelines, little importance was given in these guidelines to synovial fluid biomarkers because their use is not practical at this time.

The ICM 2018 and EBJIS criteria retain a central role in the diagnosis of PJIs in daily clinical practice; however, they might be further improved by a more detailed synovial fluid analysis (Table 3).

Some synovial biomarkers—i.e., AP, LE and CRP—are included in the ICM criteria, and therefore should be assessed when major criteria for PJI diagnosis are not present. More recent papers have confirmed P and LE accuracy while contrasting data have been recently reported regarding CRP.

Interestingly, a recent meta-analysis by Ahmad et al. [14] demonstrated that the AD test kit provides similar results compared to the ELISA laboratory test; hence the laboratory-based AD test should be preferred in daily clinical practice.

LE synovial strip test recently proved to be a reliable tool in diagnosing the persistence of PJI since it outperformed the serum CRP and ESR assays [19]. Therefore, the synovial LE strip test provides a valuable intraoperative diagnostic during the second-stage revision for PJI.

Concerning synovial IL-6, recent papers [20,21,22] have demonstrated that it should be assessed if serum IL-6 is increased, but its assessment cannot be useful in ruling out a PJI.

Some emerging synovial fluid biomarkers have been studied in recent years to better identify and treat PJIs. Among these biomarkers, calprotectin and neopterin seem to be the two most useful tools that could improve PJI diagnosis [23,24,25,26,27,28,29].

In a recent meta-analysis, calprotectin has shown a high sensitivity and specificity in the diagnosis of PJI [28]. Moreover, the CalFAST rapid test revealed a similar sensitivity and specificity, compared to the CPT ELISA test, thus it could be useful in quickly confirming or ruling out PJI [25]. In short, the daily use of calprotectin in clinical practice, as an adjunct to the ICM 2018 and EBJIS 2021 criteria, could hopefully improve the diagnosis of PJI.

Neopterin is another emerging biomarker that has shown promising results in the diagnosis of PJIs; however, further studies with a bigger sample size are needed to better define the role of this biomarker in daily clinical practice.

All the findings about emerging synovial fluid biomarkers described in the present paper are still limited to the research field and further studies and consensus are needed to apply these data to daily clinical practice. Remarkably, the routine use of SF analysis is limited by costs and lab-scientific competencies; thus, the development and validation of rapid test kits are essential to bringing the most promising innovations produced by preclinical and clinical research to the bedside.

We strongly recommend the use of the ICM 2018 and EBJIS criteria in daily clinical practice and, in the research field, the study of validated and emerging synovial fluid biomarkers to improve accuracy in the diagnosis of PJIs in daily clinical practice.

## 6. Conclusions

The routine use of the discussed synovial fluid biomarkers might improve the diagnosis of PJIs shortly. In the present review, we summarized the state-of-the-art synovial fluid biomarkers, highlighting the limits and potentials of their uses both in research and in daily clinical practice.

Further studies and consensus statements will define the roles of these emerging biomarkers in the diagnostic work of PJIs.

## Figures and Tables

**Table 1:** 2018 ICM criteria for PJI definition.

MAJOR CRITERIA		DECISION
Two positive cultures of the same organism		INFECTED (at least one of the following is present)
Sinus tract evidence of communication to the joint or visualization of the prosthesis		
**MINOR CRITERIA**	**SCORE**	**DECISION**
Elevated serum CRP or D-dimer	2	≥6, infected 2–5, possibly infected 0–1, not infected
Elevated serum ESR	1	
Elevated synovial WBC count or LE	3	
Positive synovial alpha-defensin	3	
Elevated synovial PMN (%)	2	
Elevated synovial CRP	1	

CRP = C-Reactive Protein; ESR = Erythrocyte Sedimentation Rate; WBC = White Blood Cells; LE = Leucocyte Esterase; PMN = Polymorphonuclear Cells.

**Table 3:** Synovial biomarkers features.

Biomarker	Features	Diagnostic Value
Alpha-Defensin (AD)	An elevated synovial AD is a minor criterium for PJI diagnosis, according to ICM 2018	signal-to-cut-off ratio > 1
Leukocyte Esterase (LE)	An elevated synovial fluid LE is a minor criterium to define PJIs, according to the 2018 ICM	2+ cut-off
C-Reactive Protein (CRP)	A synovial CRP concentration > 6.9 mg/L is a minor criterion for PJI diagnosis, according to ICM 2018 (but its diagnostic utility has been debated in recent years)	>6.9 mg/L
Interleukin-6 (IL-6)	Synovial IL-6 should be assessed if serum IL-6 is increased [21], but its assessment could be not useful to rule out a PJI	>2300 pg/mL
Calprotectin (CPT)	Synovial CPT is a valuable biomarker as it provides a reliable and rapid diagnosis of PJI	>50 mg/L
Presepsin (PS)	Synovial PS usefulness in the diagnosis of PJIs is a matter of debate	>1262.0 pg/mL
Neopterin (NPT)	Synovial NPT could be a reliable diagnostic marker for the detection of PJI	>7.2 nmol/L
